# Diffuse leptomeningeal gliomatosis initially presenting with intraventricular hemorrhage: a case report and literature review

**DOI:** 10.1186/s12883-015-0341-1

**Published:** 2015-05-10

**Authors:** Min Zhu, JunJun Zheng, Yuanzhao Zhu, Hui Wan, Yuchen Wu, Daojun Hong

**Affiliations:** Department of Neurology, The First Affiliated Hospital of Nanchang University, Yong Wai Zheng Street 17#, Nanchang, 330006 P.R. China

**Keywords:** Leptomeningeal gliomatosis, Hemorrhage, Anaplastic astrocytoma, Enhanced MRI, Glial fibrillary acidic protein

## Abstract

**Background:**

Primary diffuse leptomeningeal gliomatosis (PDLG) is a lethal neoplasm that is characterized by glioma cells exclusively infiltrating into cerebral and spinal meninges. Intraventricular hemorrhage as an initial symptom in PDLG patient has not been reported in the literatures.

**Case presentation:**

A 39-year-old man initially presented with intraventricular hemorrhage. The patient had an improved outcome at the early stage of hemorrhagic course; however, the clinical condition began to a sudden turn for deterioration with intracranial hypertension and cerebral hernia on day 15 after admission. Cerebral CT and MRI showed diffuse patchy signals with enhancement in bilateral cerebellopontine angle cistern, suprasellar cistern, ambient cistern, quadrigeminal cistern, bilateral cerebellum, cerebral hemisphere, and upper cervical cord surface. Pathological examination revealed that numerous spindled cells were scant of cytoplasm with hyperchromatic nuclei and various mitotic figures. Immunohistochemistry showed that the cells were positive to glial fibrillary acidic protein (GFAP) with about 5 % Ki-67 positive labeling. The pathological findings were consistent with the diagnostic criteria of anaplastic astrocytoma (WHO grade III).

**Conclusion:**

We reported an interesting case that PDLG initially presented with intraventricular hemorrhage that might be caused by astrocytoma rupturing into pial vessels.

**Electronic supplementary material:**

The online version of this article (doi:10.1186/s12883-015-0341-1) contains supplementary material, which is available to authorized users.

## Background

Primary diffuse leptomeningeal gliomatosis (PDLG) is a lethal neoplasm that is characterized by glioma cells diffusely infiltrating into cerebral and spinal meninges, while absent of tumorous lesions in the brain parenchyma [1–3]. Non-specifically insidious headache is the most common initial symptom in patients with PDLG [4]. Laboratory investigations and radiological examinations are absent of characteristic features for the diagnosis of PDLG. Therefore, it is difficult to make a definitive diagnosis during the patient’s survival time [5]. Up to date, there are about 50 cases of PDLG having been reported in literatures, and more than half of patients are made a definitive diagnosis through postmortem [2, 6, 7].

In a cohort of study, 4.9 % patients discharged with diagnosis as brain neoplasm were initially considered as a stroke [8]. About 5 % patients with gliomas would have stroke-like hemorrhage [9]. Generally, patients with tumor-related cerebral hemorrhage usually have an obvious parenchymal lesion in the brain [10]. However, no patients with PDLG initially presenting with intraventricular hemorrhage have been reported in the literatures. Herein, we described a case of 39-year-old man initially presented with intraventricular hemorrhage, finally was diagnosed as PDLG associated with anaplastic astrocytoma.

## Case presentation

**Fig. 1:**
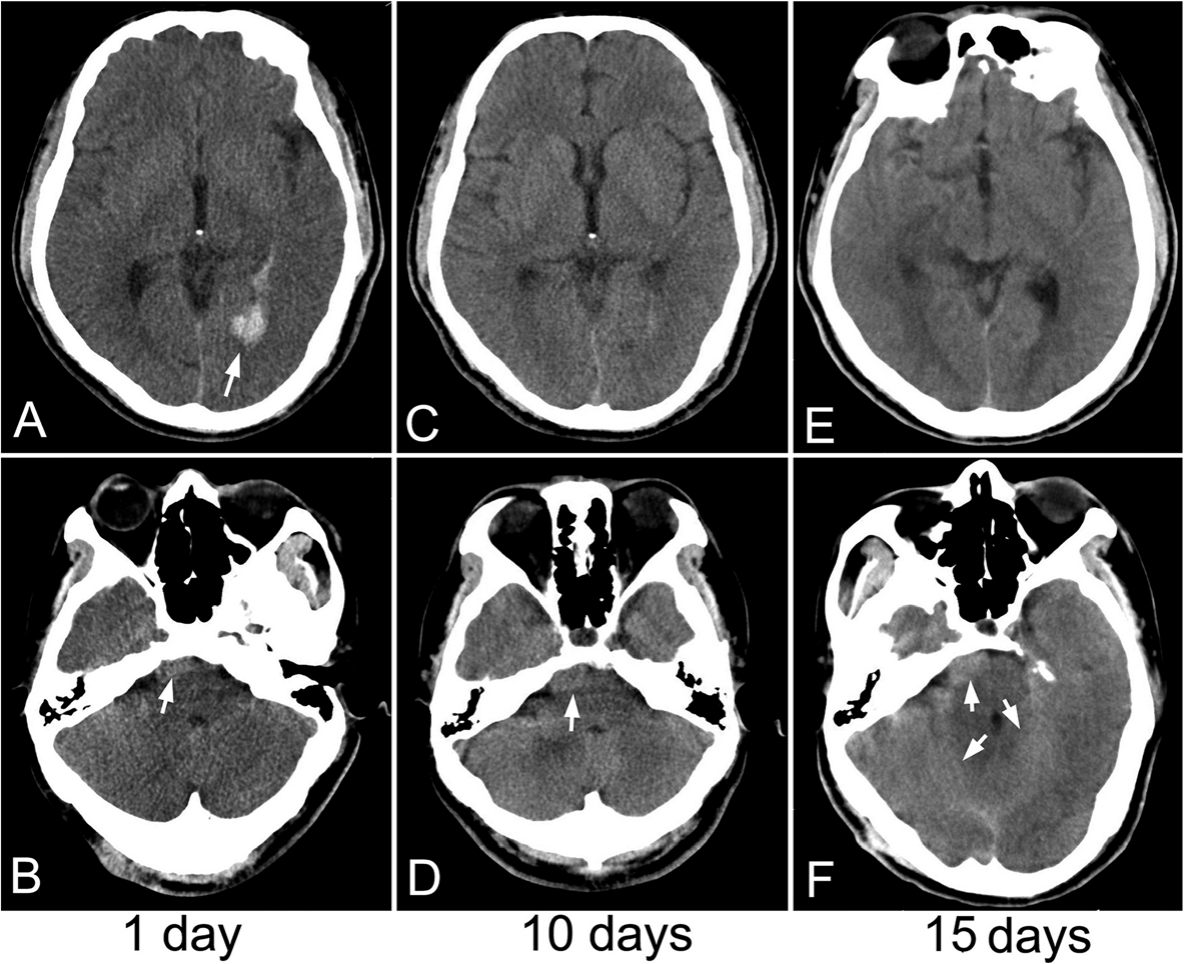
The dynamic change of cerebral CT after cerebral hemorrhage. At the first day, CT revealed hemorrhage in the left posterior horn of lateral ventricle (**a**, arrow), and a nodular mass with slightly high density at the right ventral surface of the pons (**b**, arrow). At the 10th day, CT scan showed hemorrhage almost disappeared (**c**), but the nodular mass still was unchanged (**d**, arrow). At the 15th day, CT showed hematoma was completely absorbed (**e**), but the nodular mass became more prominent accompanying abnormal density in the tentorium of cerebellum (**f**, arrow)

The patient was a 39-year-old man who was diagnosed as tuberculous pleurisy at age 30, and then was treated with standard anti-tubercular drugs for 6 months, but clinical follow-up was not carried out after alleviation of his symptoms. No history of hypertension was recorded. He had nonspecific headache one month ago, but no attentions were paid. He was admitted to our hospital with right paresis, headache and vomiting for one hour. On admission, his blood pressure was 130/80 mmHg. He had slightly slurred pronunciation; muscle strength was 4/5 grade (MRCS, grades 0–5) in the right limbs; and slight hearing loss was found on the right side. Neck stiffness and Kerning sign were also observed. Cerebral computed tomography (CT) revealed hemorrhage in the left posterior horn of lateral ventricle and subarachnoid space (Fig. 1a). At the same time, a nodular mass with slightly high density was observed at the right ventral surface of the pons (Fig. 1b). After treatments for decreasing intracranial pressure by mannitol and anti-vasospasm by nimodipine for 10 days, the patient felt great alleviation of headache. CT scan showed hemorrhage almost disappeared (Fig. 1c), but the nodular mass still was unchanged (Fig. 1d). Cerebral CT angiography underwent on day 2 after admission revealed no aneurysm and vascular malformation, but there were obvious enhancement of lesions at the right ventral surface of the pons, right temporal tip, and tentorium of cerebellum (Additional file 1: Figure S1).

**Fig. 2:**
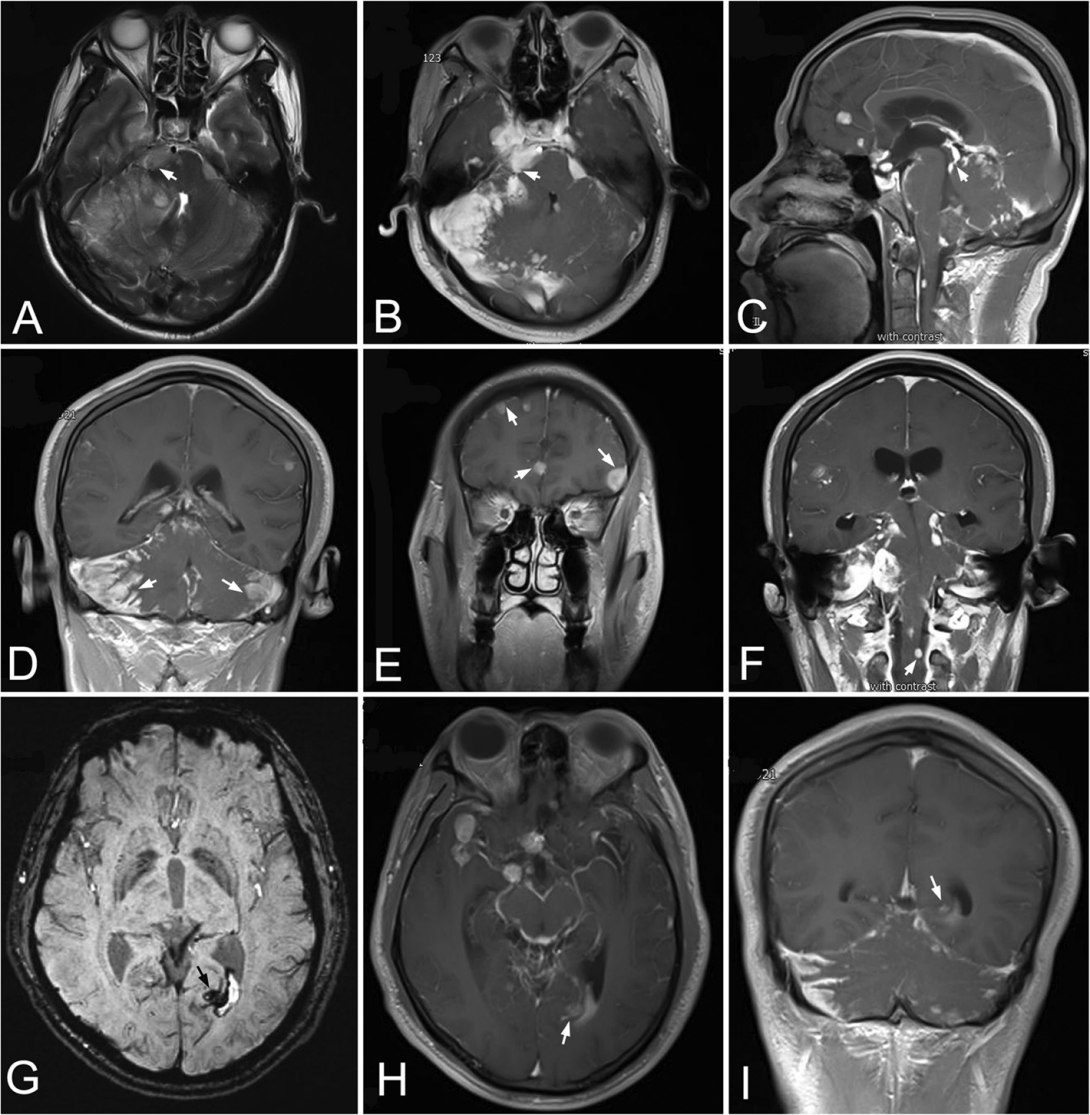
The features of cerebral MRI. MRI showed multiple abnormal patchy signals with marked enhancement in bilateral cerebellopontine angle cistern (**a**, **b**, arrow), quadrigeminal cistern (**c**, arrow), bilateral cerebellum (**d**, arrow), cerebral hemisphere (**e**, arrow), and upper cervical cord surface (**f**, arrow). SWI showed the hemorrhagic lesion located at the in-depth of left calcarine fissure (**g**, arrow), which was confirmed by the axial (**h**, arrow) and coronal (**i**, arrow) enhanced MR imaging

However, on day 15 after admission, he suddenly suffered from severe headache and recurrent vomiting. No fever and bellyache was observed. To exclude the possibility of rehemorrhage, cerebral CT was performed again. No hematoma was found (Fig. 1e), but the nodular mass became more prominent accompanying abnormal density in the tentorium of cerebellum (Fig. 1f). Cerebral magnetic resonance imaging (MRI) revealed multiple abnormal patchy signals with marked enhancement distributing in bilateral cerebellopontine angle cistern (Fig. 2a, b), suprasellar cistern, ambient cistern, quadrigeminal cistern (Fig. 2c), bilateral cerebellum (Fig. 2d), cerebral hemisphere (Fig. 2e), and upper cervical cord surface (Fig. 2f). Susceptibility weighted imaging (SWI) showed the hemorrhagic lesion located at the in-depth of left calcarine fissure (Fig. 2g), which was confirmed by the axial (Fig. 2h) and coronal (Fig. 2i) enhanced MR imaging. Magnetic resonance spectroscopy revealed that the peak of N-acetylaspartate decreased, while the peak of choline was significantly elevated. Based on the clinical features and radiological changes, several possible diseases such as tuberculous meningitis, fungal infections, meningeal neoplasm, metastases, and vasculitis were taken into consideration. So a series of examinations were further carried out. Cerebrospinal fluid (CSF) examination revealed that the number of the nucleated cells was 4 cell/ul; glucose level was 3.5 mmol/L (concomitant blood glucose 4.7 mmol/L); chlorine level was 115 mmol/L; and protein level was 2343 mg/L. Additional CSF examinations including latex agglutination test, ink stain, and modified acid fast staining were negative. Many neutrophils without malignant cells were observed in CSF cytology. No neoplasm was found by thoracoabdominal CT. Positron emission tomography (PET) scan showed an increased uptake in the right parietal lobe, left frontal lobe, right temporal lobe, and bilateral cerebellum. Laboratory examinations revealed serum tumor biomarkers (AFP, CEA, CA199, CA125, CA153, CA724, PSA, NSE and Cyfra21-1), blood routine, thyroid function, extractable nuclear antigen polypeptide spectrum, and anti-neutrophil cytoplasmic antibody were normal. T-spot test was positive. After tuberculous meningoencephalitis and fungal infections were excluded, intravenous dexamethasone (20 mg for 7 days and then 10 mg for 3 days) was started on day 20 after admission, but no clinical improvement was observed.

On day 36 after admission, second CSF examination was performed. Results showed nucleated cells count was 8 cells/uL; glucose level was 3.39 mmol/L (concomitant blood glucose 5.37 mmol/L); chlorine level was 116 mmol/L; and protein level was 1787 mg/L. Malignant cells also were not found in cytology. His situation showed no signs of improvement and gradually worsened daily. After a written consent signed by patient’s wife, a needle biopsy of the lesion in the left frontal lobe was performed on day 46 after admission. On day 3 after biopsy, he died from cerebral hernia owing to intractable intracranial hypertension.

**Fig. 3:**
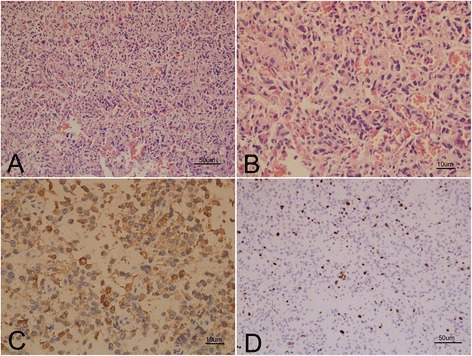
The Pathological changes of the lesion at the bottom of right frontal lobe. Hematoxylin eosin (HE) stain revealed numerous tumor cells were scant of cytoplasm with hyperchromatic nuclei and various mitotic figures (**a,** bar=50um), high magnification (**b**, bar=10um). Immunohistochemistry showed tumor cells were positive to glial fibrillary acidic protein (GFAP) (**c**, bar=10um). Ki-67 labeling indicated about 5 % index of proliferation (**d**, bar=50um)

Pathological examination revealed that numerous spindled cells were scant of cytoplasm with hyperchromatic nuclei and various mitotic figures (Fig. 3a, b). Necrosis and vascular endothelial proliferation were not observed. Immunohistochemistry showed that the tumor cells were positive to glial fibrillary acidic protein (GFAP) (Fig. 3c) and S-100 protein. The timorous lesion was negative to CD34, epithelial membrane antigen, progesterone receptor, and vimentin. Ki-67 labeling indicated about 5 % index of proliferation (Fig. 3d). The pathological findings were consistent with the diagnostic criteria of anaplastic astrocytoma (WHO grade III).

## Discussion

Leptomeningeal gliomatosis is usually divided into secondary type and primary type [11]. The secondary type is caused by metastasis of glioma originated in the brain parenchyma; however, the primary type may arise from heterotopic glial cell nests in the pial surface of the brain and spinal cord, which can be found throughout the neuroaxis [12]. Prior to high resolution imaging, the definitive diagnosis of PDLG was dependent on autopsy to rule out a primary parenchymal source for the leptomeningeal seeding [6, 7, 13]. With the advent of improved imaging techniques, the diagnosis of PDLG is becoming more common and can be legitimately considered during the patient’s survive time, especially based on the gadolinium-enhanced MRI and biopsy [14, 15]. Cerebral MRI in our patient revealed diffuse, nodular leptomeningeal enhancement around the surface of brainstem, cerebellum, cerebrum, and upper cervical cord, while absent of distinct parenchymal lesions on the MR imaging. Ultimately, the diagnosis of PDLG was established on the pathological results.

The most interesting clinical feature was intracranial hemorrhage as the initial symptom in this PDLG patient. Burrus TM *et al.* in 2009 described a case diagnosed as hemorrhagic glioblastoma of the frontal lobe adherent to the underlying dura with leptomeningeal metastases [16]. However, intraventricular hemorrhage was first described in our patient with diffuse leptomeningeal lesions. The natural history of PDLG contains two stages including chronic prodromal phase and acute neurological deficits [1–3]. The main clinical manifestations may include headache, papilledema, cranial never paralysis, seizure, back pain, paresthesia, decreased mental status, and visual impairment, which are nonspecific and difficult to make a definite diagnosis at the early stage [4, 15]. As for this patient, we initially made a misdiagnosis as simple cerebral hemorrhage after ruling out aneurysm and vascular malformation. In fact, the clinical condition of this patient was gradually improved in accordance with the outcome of cerebral hemorrhage during the first stage of clinical course. However, the patient’s condition suddenly became worse when the ruptured blood completely disappeared. The clinical shift, rapid aggressiveness, and short course of this disease were completely beyond our expectations. It still needs to further discuss whether intracranial hemorrhage can promote the dissemination and tumor seeding of leptomeningeal gliomatosis.

The most important features of PDLG in radiological images are exclusive lesions in pia mater, which are prone to involving in posterior fossa, basilar cistern and spinal cord [14, 15]. Cerebral MRI in our case showed multiple nodular lesions in brain and upper cervical cord indicating the tumor widely distributing in CNS, which was conformed to the diffuse type of PDLG [17]. Many of the cases reported as PDLG are complicated by focal parenchymal involvement as our case, which have been interpreted as a secondary parenchymal infiltration of a primary leptomeningeal glioma [18]. However, this distinction of primary and secondary leptomeningeal glioma is impossible to differentiate only by radiological changes. Thus, the literatures on PDLG are confused with many cases that could likely represent a secondary meningeal leptomeningeal gliomatosis [18, 19]. A nodular mass with high density was found on the surface of right pons when we retrospectively analyzed the first cerebral CT. The lesions were clearer in the enhanced CT image performed at the next day of the first CT. The above findings would rule out the possibility of parenchymal gliomatosis seeding into the meningeal system through the hemorrhage. As for the hemorrhagic lesion, the MRI showed the lesion located between the in-depth calcarine fissure and posterior horn of lateral ventricle. The enhanced MRI clearly revealed leptomeningeal gliomatosis infiltrating into the hemorrhagic lesion.

The pathological features in all of PDLG patients were almost consistent with high-grade gliomas (WHO III or IV) [1, 2]. In our case, the lesion showed the proliferation of tumor cells with moderate intensity, absent of necrosis and vascular endothelial proliferation. Tumor cells had significant pleomorphism and atypia accompanying GFAP expression and 5 % index of Ki-67 labeling. All the findings were consistent with the pathological features of anaplastic astrocytoma (WHO Grade III). Saito R. *et al.* reported patients with leptomeningeal dissemination of anaplastic astrocytoma suffered from subarachnoid hemorrhage after radiochemotherapy [20]. The hemorrhagic reasons might be associated with vessels damage by radiochemotherapy, or with tumor cells rupturing into pial vessels. Therefore, our patient with PDLG presenting with intraventricular hemorrhage that might be caused by astrocytoma cells rupturing into pial vessels.

This diagnosis should be differentiated from several diseases. First of all, the tubercular meningitis should be considered. Antituberculosis therapy had been administered in more than half of reported cases with PDLG [21]. Our patient had a history of tuberculosis infection, positivity of T-SPOT examination, and aggregated lesions on cranial basis. However, he had no tubercular toxic symptoms, negativity of tubercle bacillus stain, as well as normal level of glucose and chlorine in twice CSF examinations. All of those evidences did not support the diagnosis of tubercular meningitis. Second, the patient presented with chronic headache, intracranial hypertension, and lesions mainly involving pia mater, which led to suspect the diagnosis of fungal meningitis. There was a report of cryptococcal meningitis manifesting as pseudo-subarachnoid hemorrhage [22]. However, the glucose level was normal, India ink stain and latex agglutination tests were negative in twice CSF examinations. Third, malignant melanoma should also be considered because of its high incidence of hemorrhage in central nervous system [23]. However, cutaneous melanoma was not found in the patient. In addition, different MR sequences were not fully consistent with the features of malignant melanoma. Fourth, meningeal carcinomatosis should be also taken into consideration. However, no latent cancers were identified in the whole body through tumor biomarker examinations, thoracic-abdominal CT, and PET scan. Finally, cerebral vasculitis should be distinguished. Glioblastoma mimicking as cerebral vasculitis was reported in the literature [24].

## Conclusion

In summary, we described a case of primary diffuse leptomeningeal gliomatosis associated with anaplastic astrocytoma. It was an interesting case of PDLG initially presenting with intraventricular hemorrhage that might be caused by astrocytoma rupturing into pial vessels.

### Patient consent

Written informed consent was obtained from the patient’s wife for publication of this case report and any accompanying images. A copy of the written consent is available for review by the editor of this journal.

## Additional file

Additional file 1:
**The supplemental figure presented with the features of cerebral CT angiography.**

## Abbreviations

PDLG: Primary diffuse leptomeningeal gliomatosis
CT: Cerebral computed tomography
MRI: Cerebral magnetic resonance imaging
SWI: Susceptibility weighted imaging
CSF: Cerebrospinal fluid
GFA P: Glial fibrillary acidic protein
