# Supplementary material for: Diffuse leptomeningeal gliomatosis initially presenting with intraventricular hemorrhage: a case report and literature review
Source: BMC Neurol. 2015 May 10;15:77. doi: 10.1186/s12883-015-0341-1 (PMC4489354; doi:10.1186/s12883-015-0341-1)
Supplement: Additional file 1: — The supplemental figure presented with the features of cerebral CT angiography. [file 12883_2015_341_MOESM1_ESM.pdf]

Title page (additional file)

Diffuse leptomeningeal gliomatosis initially presenting with intraventricular hemorrhage: a case report and literature review

Min Zhu, JunJun Zheng, Yuanzhao Zhu, Hui Wan, Yuchen Wu, Daojun Hong

Department of Neurology, The First Affiliated Hospital of Nanchang University, China

Email address: Min Zhu: zhumin1@126.com; JunJun Zheng: zjj259@163.com; Yuanzhao Zhu: 846366855@qq.com; Hui Wan: ncwanhui@sina.com; Yuchen Wu: wuyuchen52@163.com; Daojun Hong: hongdaojun@hotmail.com

Corresponding author:

Dr. Daojun Hong

Department of Neurology, The First Affiliated Hospital of Nanchang University

Yong Wai Zheng Street 17#, Nanchang, 330006, P.R.China

Telephone: 86-791-8869-2511

Fax number: 86-791-8869-2511

E-mail: hongdaojun@hotmail.com

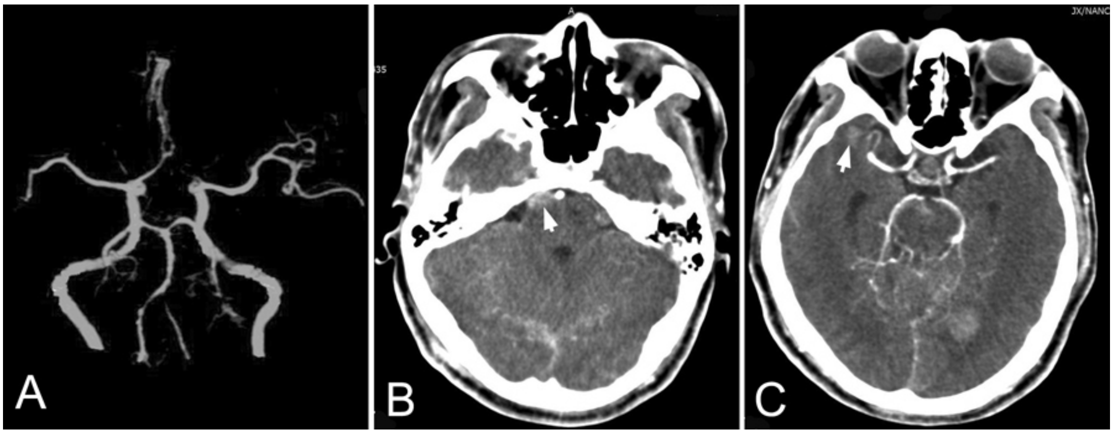

Figure 1. The features of cerebral CT angiography. No aneurysm and vascular malformation were found (A). Multiple lesions with enhancement at the right ventral surface of the pons (B, arrow), and right temporal horn (C, arrow) were observed.
